# Clinicopathological Evaluation of Terminal Ileal Nodular Lymphoid Hyperplasia in Adults: A Single-Center Experience

**DOI:** 10.3390/jcm15218761

**Published:** 2026-11-09

**Authors:** Ümit Karabulut, Ahmet Yavuz, Çiğdem Budak Ece, Pınar Ergen

**Affiliations:** 1 Department of Gastroenterology, University of Health Sciences Diyarbakır Gazi Yaşargil Training and Research Hospital, 21070 Diyarbakır, Turkey; 2 Department of Pathology, University of Health Sciences Diyarbakır Gazi Yaşargil Training and Research Hospital, 21070 Diyarbakır, Turkey

**Keywords:** terminal ileum, nodular lymphoid hyperplasia, *Giardia lamblia*, fecal calprotectin, Crohn’s disease

## Abstract

**Background/Objectives:** Terminal ileal nodular lymphoid hyperplasia (NLH) can mimic Crohn’s disease endoscopically, and data integrating its clinical, laboratory, endoscopic, and histopathological features in adults are limited. We aimed to describe these features and associated conditions. **Methods:** This retrospective single-center study included adults who underwent colonoscopy between January 2023 and December 2025 with terminal ileal biopsy for endoscopically suspected NLH; histopathological confirmation was required for inclusion. *Giardia lamblia* (*G. lamblia*)–positive and –negative patients were compared as a secondary exploratory analysis. **Results:** Histology confirmed NLH in 50 of 64 biopsied patients (78.1%); 43 with essential inclusion data were analyzed (mean age 35.1 ± 12.0 years; 67.4% male); fecal calprotectin was available in 40. Diarrhea and abdominal pain were leading indications. Findings were predominantly reactive, with no morphological features of lymphoma; one patient with Crohn’s-suggestive features was subsequently diagnosed with Crohn’s disease. No patient had hypogammaglobulinemia or positive celiac serology. *G. lamblia* infection (10/43, 23.3%) was associated with higher fecal calprotectin (median 36.5 vs. 9.0 µg/g; *p* = 0.016). **Conclusions:** On routine histology, adult terminal ileal NLH appears predominantly benign and reactive; however, endoscopic appearance alone was insufficient for diagnosis in one fifth of cases. The *G. lamblia*–calprotectin association is exploratory and requires confirmation.

## 1. Introduction

Nodular lymphoid hyperplasia (NLH) is a benign lymphoid proliferation characterized by numerous small nodules, typically ranging from 2–10 mm in diameter, within the gastrointestinal tract. Although it can also occur in the stomach, colon, and rectum [1], it is most frequently observed in the small intestine, particularly the terminal ileum. Histologically, NLH is characterized by well-circumscribed lymphoid follicles with prominent germinal center formation and a high mitotic rate.

The clinical presentation of terminal ileal NLH is usually nonspecific and may include abdominal pain and diarrhea. Endoscopically, its appearance as multiple nodular lesions can create diagnostic difficulty in distinguishing it from Crohn’s disease–related terminal ileitis, particularly in symptomatic adults [2,3]. However, terminal ileal nodularity is not specific to NLH and may also reflect physiological lymphoid prominence, particularly in younger individuals, or infectious, inflammatory, or lymphoproliferative conditions [1]. Histopathological confirmation is therefore essential for accurate diagnosis.

Although the etiopathogenesis of NLH has not been fully elucidated, chronic antigenic stimulation and immune dysregulation are thought to play a role in its development. In adults, NLH has been reported in association with common variable immunodeficiency (CVID), selective IgA deficiency, *Giardia lamblia* (*G. lamblia*) infection, and celiac disease; it has also been linked to the development of lymphoma [4]. Nevertheless, a substantial proportion of patients with NLH show no identifiable underlying etiological cause.

The existing literature on ileal NLH consists predominantly of case reports and small series [3,5], with limited studies evaluating clinical, laboratory, endoscopic, and histopathological features together in the same patient population. This gap represents a significant source of uncertainty for clinicians encountering nodular lesions in daily practice: how often this appearance is encountered, how much clinical concern it should raise, and whether routine biopsy is warranted remain unclear.

In this study, we aimed to contribute to the recognition of this clinically underdefined entity by retrospectively describing the demographic, clinical, laboratory, endoscopic, and histopathological features of a series of adult patients with histopathologically confirmed terminal ileal NLH. As part of this evaluation, conditions previously reported in association with NLH, including *G. lamblia* infection, celiac disease, and immunodeficiency, were also screened for.

## 2. Materials and Methods

### 2.1. Study Design and Patient Selection

This retrospective, single-center observational study was conducted at the Department of Gastroenterology, University of Health Sciences Diyarbakır Gazi Yaşargil Training and Research Hospital. The records of patients aged ≥18 years who underwent colonoscopy for any indication between January 2023 and December 2025 were reviewed retrospectively through the hospital’s electronic data management system. Consecutive patients meeting the eligibility criteria were included; for patients who underwent more than one colonoscopy during the study period, only the first examination with terminal ileal biopsy was considered.

Patients in whom colonoscopy reports described an endoscopic appearance consistent with NLH—defined as multiple small nodular lesions approximately 2–10 mm in diameter in the terminal ileum—and who consequently underwent terminal ileal biopsy were included in the evaluation. Terminal ileal biopsies were obtained using standard biopsy forceps. The number of biopsy specimens was not standardized across procedures and was left to the endoscopist’s clinical judgment.

In accordance with previously established endoscopic and histopathological criteria for NLH [1,3,6], the diagnosis in this study was considered clinicopathological, requiring both a compatible endoscopic appearance (multiple small nodular lesions in the terminal ileum) and histopathological confirmation. A diagnosis of NLH was established when histopathological examination demonstrated well-circumscribed, hyperplastic lymphoid follicles with prominent germinal center formation. Patients with an established diagnosis of Crohn’s disease, ulcerative colitis, intestinal tuberculosis, malignancy, or lymphoma at the time of the index colonoscopy, or whose index biopsy findings were diagnostic of any of these or of another specific inflammatory or neoplastic disease, were excluded. Isolated histological features that were suggestive of, but not diagnostic for, a specific disease (e.g., focal granulomatous inflammation) did not constitute grounds for exclusion; such patients were retained in the analysis and are reported separately. To minimize potential overlap with an isolated Peyer’s patch, NLH was diagnosed only when hyperplastic follicles were identified in multiple, non-contiguous biopsy fragments corresponding to the diffusely nodular endoscopic appearance, rather than being confined to a single fragment. Patients with insufficient essential laboratory data were also excluded from the final analysis; essential data were defined as the core etiological work-up, namely serum immunoglobulin levels and stool microscopy for intestinal parasites. Fecal calprotectin was not considered an inclusion requirement, and patients with missing fecal calprotectin data were retained in the analysis.

### 2.2. Data Collection

Patients’ demographic characteristics, colonoscopy indications, endoscopic findings, histopathological results, laboratory parameters, and clinical features were obtained from the electronic medical record system.

Variables assessed included age, sex, indication for colonoscopy, endoscopic appearance of the terminal ileum, complete blood count, biochemical parameters, C-reactive protein (CRP), immunoglobulin levels (IgG, IgA, IgM, and IgE), celiac serology, fecal calprotectin level, stool examinations for *Giardia lamblia* and other intestinal parasites, smoking status, and history of immunosuppressive therapy. Because terminal ileal nodularity can mimic Crohn’s disease and has been associated with immunodeficiency states, *Giardia lamblia* infection, and celiac disease in the literature [1,2,3,^7^,^8^,^9^,^10^], immunoglobulin levels, celiac serology, fecal calprotectin, fecal occult blood testing, and stool examination for intestinal parasites were routinely requested for all patients with an endoscopic appearance of terminal ileal lymphoid hyperplasia as part of a standardized etiological work-up, rather than being selectively ordered based on individual clinical indications. In this study, these conditions are referred to as associated conditions; their presence indicates co-occurrence with NLH and does not imply an established causal role. However, owing to the retrospective design, results were not available for every patient (e.g., tests not performed or results not retrievable from the electronic records); the number of patients with available data is therefore reported for each parameter in the Results section. *G. lamblia* (syn. *G. duodenalis*, *G. intestinalis*) infection was diagnosed by a rapid immunochromatographic stool antigen test performed on a single stool sample, with a positive result defined according to the manufacturer’s instructions; other intestinal parasites were assessed by stool microscopy; celiac serology was assessed by tissue transglutaminase IgA (tTG-IgA), with values >10 U/mL considered positive; and fecal calprotectin was measured using an enzyme-linked immunosorbent assay (ELISA), with values >50 µg/g considered elevated in accordance with the widely used cut-off for distinguishing intestinal inflammation [11].

### 2.3. Study Population

During the study period, a total of 64 patients underwent biopsy for an endoscopic appearance consistent with NLH in the terminal ileum. Histopathological examination confirmed a diagnosis of NLH in 50 of these patients (78.1%); this figure represents the proportion of endoscopic suspicions of NLH confirmed by histology. In the remaining 14 patients (21.9%), histopathological examination did not demonstrate hyperplastic lymphoid follicles fulfilling the multifocal, non-contiguous pattern required for NLH diagnosis (see Section 2); biopsies from these patients showed unremarkable mucosa or findings consistent with an isolated Peyer’s patch, without a specific alternative diagnosis, and they were therefore excluded from further analysis. Of the 50 histopathologically confirmed NLH patients, 7 were excluded because of missing essential immunological and parasitological data (serum immunoglobulin levels and stool microscopy); fecal calprotectin was also unavailable in these seven patients but was not a criterion for exclusion. The final analysis therefore comprised 43 patients (Figure 1). All 43 patients had complete data for stool *G. lamblia* antigen testing, stool microscopy, serum immunoglobulin levels, and celiac serology; fecal calprotectin was available in 40 patients (10 of 10 *G. lamblia*–positive and 30 of 33 *G. lamblia*–negative patients), and the calprotectin comparison was therefore performed in these 40 patients.

### 2.4. Ethical Approval

The study was approved by the Clinical Research Ethics Committee of the University of Health Sciences Diyarbakır Gazi Yaşargil Training and Research Hospital (Decision No: 010, Date: 9 January 2026) and was conducted in accordance with the principles of the Declaration of Helsinki.

### 2.5. Histopathological Evaluation

Initial histopathological diagnoses were made in routine clinical practice by the on-duty gastrointestinal pathologist at the time of biopsy. For the purposes of this study, all hematoxylin and eosin (H&E)–stained sections were subsequently re-reviewed by a single experienced pathologist, blinded to patients’ clinical and laboratory data, to confirm the diagnosis and ensure uniform application of the histopathological criteria described above. The 14 patients excluded for lack of histopathological confirmation of NLH were reviewed by this same pathologist during the same re-evaluation process; because re-evaluation was performed by a single pathologist, interobserver agreement could not be assessed. Follicles were considered reactive when they showed preserved follicular architecture with polarized germinal centers containing tingible body macrophages, an intact mantle zone of small mature lymphocytes, and a morphologically heterogeneous (polymorphous) cell population, in the absence of follicular effacement, a monotonous atypical lymphoid infiltrate, or lymphoepithelial lesions [1,3,6]. Features suggestive of Crohn’s disease were defined as chronic architectural distortion (crypt branching or loss, villous blunting), basal lymphoplasmacytosis, pyloric gland metaplasia, and non-caseating epithelioid granulomas. In addition to lymphoid follicles, crypt architecture, villous structure, and the presence of granulomatous and active inflammation were assessed; histopathological findings suggestive of malignancy, lymphoma, or inflammatory bowel disease were also recorded. All evaluations were based on routine H&E morphology; immunohistochemistry and clonality analyses were not performed.

### 2.6. Statistical Analysis

Statistical analyses were performed using IBM SPSS Statistics for Windows, Version 26.0 (IBM Corp., Armonk, NY, USA). The normality of continuous variables was assessed using the Shapiro–Wilk test. Normally distributed continuous variables are presented as mean ± standard deviation, and non-normally distributed variables as median (interquartile range or minimum–maximum). Categorical variables are expressed as number (*n*) and percentage (%).

Comparisons between groups for normally distributed continuous variables were performed using the independent-samples Student’s t-test, and for non-normally distributed continuous variables using the Mann–Whitney U test. Categorical variables were compared using the Pearson chi-square test, or Fisher’s exact test when the expected cell frequency was below 5. All statistical tests were two-tailed, and a *p*-value < 0.05 was considered statistically significant. Subgroup comparisons (e.g., between *G. lamblia*–positive and –negative patients) were performed without correction for multiple comparisons; the corresponding findings should therefore be regarded as exploratory (hypothesis-generating). To assess the robustness of the main exploratory finding, a sensitivity analysis was performed after excluding patients with histological features suggestive of a specific inflammatory disease at the index biopsy. In the *G. lamblia* subgroup analysis, five variables were compared between groups (age, sex, endoscopic ileitis, nonspecific inflammation, and fecal calprotectin), and the proportion of patients with fecal calprotectin >50 µg/g was additionally compared using Fisher’s exact test.

## 3. Results

A total of 43 patients with histopathologically confirmed terminal ileal NLH were included in the study. The mean age of the patients was 35.1 ± 12.0 years, with a male predominance (67.4%). Diarrhea and abdominal pain were the most common indications for colonoscopy. Slightly over half of the patients (51.2%) were active smokers; no patient had a history of immunosuppressive therapy or a family history of inflammatory bowel disease. Demographic and clinical characteristics are summarized in Table 1.

**Table 1:** Demographic and clinical characteristics of patients with terminal ileal nodular lymphoid hyperplasia (*n* = 43).

Variable	Value
Age (years), mean ± SD	35.1 ± 12.0
Male sex, *n* (%)	29 (67.4)
Female sex, *n* (%)	14 (32.6)
Current smoking, *n* (%)	22 (51.2)
Primary indication for colonoscopy, *n* (%)	
Diarrhea	13 (30.2)
Abdominal pain	10 (23.3)
Screening	7 (16.3)
Constipation	7 (16.3)
Anemia	5 (11.6)
Lower gastrointestinal bleeding	1 (2.3)

### 3.1. Laboratory Findings

Laboratory findings are presented in Table 2. Complete blood count parameters were generally within normal reference ranges. Similarly, inflammatory markers such as C-reactive protein and erythrocyte sedimentation rate did not suggest significant systemic inflammation in the majority of patients. Serum albumin, ferritin, vitamin B12, and folate levels were also generally within reference ranges. On immunological assessment, no patient had serum IgA, IgG, or IgM levels below the lower reference limit; thus, no hypogammaglobulinemia or selective IgA deficiency was observed.

**Table 2:** Laboratory characteristics of patients with terminal ileal nodular lymphoid hyperplasia.

Parameter	Reference Range	*n*	Value
Complete Blood Count (CBC)			
White blood cell count (×10^9^/L)	4.0–10.0	43	8.4 ± 2.4
Neutrophil count (×10^9^/L)	2.0–7.5	43	5.4 ± 2.1
Lymphocyte count (×10^9^/L)	1.0–4.0	43	2.38 ± 0.72
Platelet count (×10^9^/L)	150–400	43	272 ± 67
Inflammatory Markers			
C-reactive protein (mg/L)	<5	41	1.0 (1.0–3.0)
Erythrocyte sedimentation rate (mm/h)	M: <15, F: <20	33	10 (9–15)
Fecal calprotectin (µg/g)	<50	40	9.5 (7.0–20.3)
Biochemical Parameters			
Albumin (g/L)	35–52	42	43.8 ± 4.4
Ferritin (ng/mL)	M: 30–400, F: 13–150	41	73 (20–107)
Vitamin B12 (pg/mL)	200–900	37	350 (300–400)
Folate (ng/mL)	>4.0	32	4.3 (4.0–5.0)
Immunological Parameters			
Immunoglobulin A (IgA) (g/L)	0.7–4.0	43	2.0 ± 0.7
Immunoglobulin G (IgG) (g/L)	7.0–16.0	43	11.9 ± 2.3
Immunoglobulin M (IgM) (g/L)	0.4–2.3	43	1.17 ± 0.47
Immunoglobulin E (IgE) (IU/mL)	0–100 *	33	22 (10–50)

Data are presented as mean ± standard deviation for normally distributed variables and median (interquartile range) for non-normally distributed variables. *n* indicates the number of patients with available data for each parameter. Parameters with *n* < 43 reflect tests that were not performed or whose results could not be retrieved from the electronic records. * The reference range for serum IgE may vary according to age and laboratory assay.

### 3.2. Endoscopic and Histopathological Findings

Endoscopically, the nodules were typically small, round to oval, whitish to pale pink in color, with a smooth surface, without ulceration or friability of the nodules themselves. Endoscopic and histopathological findings are summarized in Table 3. A nodular appearance consistent with NLH was present in all patients in the terminal ileum; the most common accompanying endoscopic finding was ileitis. Ulcers (*n* = 4) and erosions (*n* = 3) were observed in the surrounding ileal mucosa rather than on the nodules; all occurred in patients with endoscopic ileitis, and three of the four patients with ulcers were *G. lamblia*–positive. Histopathological examination confirmed the diagnosis of NLH in all patients by demonstrating hyperplastic lymphoid follicles, accompanied by nonspecific inflammatory changes in most cases. Granulomatous inflammation was observed in two patients: one also had histological features suggestive of Crohn’s disease (as defined in Section 2.5), which were not diagnostic on their own, and was subsequently diagnosed with Crohn’s disease on further clinical evaluation, whereas the other had nonspecific granulomatous inflammation; in neither case were the granulomas consistent with tuberculosis. In addition, no morphological features suggestive of lymphoma were identified on routine histology in any patient.

Histologically, terminal ileal NLH consisted of increased numbers of hyperplastic lymphoid follicles located within the mucosa and/or submucosa (Figure 2). Variability in follicle shape and size was observed, while tissue integrity and cellular architecture were preserved. Reactive follicles were surrounded by a well-defined “mantle zone” composed of small, mature lymphocytes.

On high-power examination, the cell population comprising the lymphoid follicles was morphologically heterogeneous (polymorphous), with centrocytes, centroblasts, immunoblasts, and histiocytes present together (Figure 3). Tingible body macrophages clearing cellular debris were observed within the germinal centers.

**Table 3:** Endoscopic and histopathological findings.

Variable	*n* (%)
Endoscopic Findings	
Nodular lymphoid hyperplasia	43 (100)
Endoscopic ileitis	11 (25.6)
Ileal ulcer	4 (9.3)
Ileal erosion	3 (7.0)
Histopathological Findings	
Hyperplastic lymphoid follicles	43 (100)
Nonspecific inflammatory changes	39 (90.7)
Eosinophilic infiltration	2 (4.7)
Granulomatous inflammation	2 (4.7)
Findings suggestive of Crohn’s disease	1 (2.3)

Data are presented as *n* (%). NLH, nodular lymphoid hyperplasia. Findings suggestive of Crohn’s disease were not diagnostic at the index biopsy; this patient was subsequently diagnosed with Crohn’s disease. Of the two patients with granulomatous inflammation, one was this patient and the other had nonspecific granulomatous inflammation; neither showed features consistent with tuberculosis. Ileal ulcers and erosions were located in the mucosa surrounding the nodules and were not observed on the nodules themselves.

### 3.3. Associated Conditions

Associated conditions are presented in Table 4. *G. lamblia* infection was detected in 10 patients (23.3%), whereas *Entamoeba histolytica* (*E. histolytica*) was identified in only two patients. The fecal occult blood test was positive in nearly half of the patients (46.5%), whereas elevated fecal calprotectin levels were less frequent (5/40, 12.5%). Celiac serology (tissue transglutaminase IgA) was available in all 43 patients and was negative in all; as no patient had serum IgA below the lower reference limit, IgA-based serology was considered interpretable.

**Table 4:** Associated conditions in patients with terminal ileal nodular lymphoid hyperplasia.

Variable	*n*/*N* (%)
Infectious factors	
*Giardia lamblia infection*	10/43 (23.3)
*Entamoeba histolytica infection*	2/43 (4.7)
Stool test findings	
Positive fecal occult blood test	20/43 (46.5)
Elevated fecal calprotectin (>50 µg/g)	5/40 (12.5)
Associated diseases	
Positive celiac serology	0/43 (0)
Histopathological findings suggestive of Crohn’s disease	1/43 (2.3)

As a secondary exploratory analysis, when *G. lamblia*–positive and –negative patients were compared, no significant differences were found between the groups in terms of age, sex, endoscopic ileitis, or nonspecific inflammation (Table 5). In contrast, fecal calprotectin levels were significantly higher in *G. lamblia*–positive (*n* = 10) than in *G. lamblia*–negative patients (*n* = 30) (median 36.5 [IQR: 9.0–57.8] µg/g vs. 9.0 [IQR: 7.0–15.0] µg/g; Mann–Whitney U test, *p* = 0.016) (Table 5; Figure 4). The proportion of patients with fecal calprotectin > 50 µg/g was also higher in *G. lamblia*–positive than in *G. lamblia*–negative patients (4/10 [40.0%] vs. 1/30 [3.3%]; *p* = 0.010). In a sensitivity analysis excluding the patient subsequently diagnosed with Crohn’s disease, the difference remained significant (*n* = 39; *p* = 0.014); it also remained significant after additionally excluding the other patient with granulomatous inflammation (*n* = 38; *p* = 0.008).

## 4. Discussion

In this study, we evaluated adult patients with terminal ileal NLH by assessing clinical, laboratory, endoscopic, and histopathological findings together within the same series. Histopathological findings predominantly consisted of reactive lymphoid follicles; changes suggestive of Crohn’s disease, granulomatous inflammation, and eosinophilic infiltration were rare. Within the limits of routine H&E-based assessment, these findings support the view that NLH is generally a benign and reactive condition. However, one patient with histological features suggestive of Crohn’s disease at the index biopsy was subsequently diagnosed with Crohn’s disease, indicating that terminal ileal nodularity may occasionally represent an early or coexisting manifestation of Crohn’s disease. NLH should therefore be regarded as predominantly, rather than uniformly, benign, and patients with suggestive histological features, such as granulomatous inflammation, warrant clinical follow-up. Importantly, the main exploratory finding remained unchanged when these patients were excluded in the sensitivity analysis.

Our clinical findings (diarrhea, abdominal pain, constipation) are consistent with previous series describing adult ileal NLH [3,5]. However, the incidental detection of some cases on screening colonoscopy also indicates that the condition may present asymptomatically, which is in line with pediatric studies describing a range of clinical presentations [12,13]. Fecal occult blood testing was positive in nearly half of the patients (46.5%), and anemia was the indication for colonoscopy in 11.6%. Gastrointestinal bleeding has been described among the presentations of NLH [1]; however, in our series the nodules themselves showed no ulceration or friability, and accompanying ileitis, ulcers or erosions, infectious factors such as *G. lamblia*, or other colonic and anorectal sources may also have contributed. Because a positive fecal occult blood test is nonspecific and other bleeding sources were not systematically evaluated, a causal relationship between NLH and occult blood loss cannot be established in this study. Nevertheless, these findings suggest that a positive fecal occult blood test or anemia in a patient with terminal ileal nodularity should prompt evaluation for accompanying mucosal inflammation and other bleeding sources rather than being attributed to NLH alone.

A notable male predominance (67.4%) was observed in our series, which contrasts with the reverse distribution (female:male ratio 2:1) reported in the classic series by Rubio-Tapia et al. [5]. This discrepancy may be attributable to differences in population characteristics or sample size; the present data are insufficient to clarify the underlying reason.

Histopathology confirmed NLH in 50 of 64 patients (78.1%) biopsied for endoscopically suspected NLH; thus, the endoscopic appearance alone did not correspond to histologically defined NLH in approximately one fifth of cases. Because terminal ileal nodularity can mimic Crohn’s disease both endoscopically and on imaging [2,3,14], histopathological confirmation remains essential for accurate differential diagnosis [6]. As NLH was defined histologically in this study, our design does not allow assessment of the diagnostic accuracy of endoscopy or histopathology for distinguishing NLH from Crohn’s disease, nor of the impact of biopsy on treatment decisions; nevertheless, these descriptive data are consistent with the routine biopsy of such nodular lesions recommended in the literature [3,6], and histological confirmation may potentially help reduce diagnostic uncertainty and support appropriate treatment decisions. Moreover, lymphoid nodularity of the terminal ileum may represent a physiological variant in children and young adults, reflecting prominent Peyer’s patches [1,14]. Although we attempted to minimize this overlap by requiring hyperplastic follicles in multiple, non-contiguous biopsy fragments, given the relatively young age of our cohort (mean 35.1 years), some cases may nonetheless represent physiological lymphoid prominence rather than pathological hyperplasia, and the two cannot be reliably distinguished on morphology alone.

The literature suggests that NLH generally resolves spontaneously and does not require specific treatment; Lin et al. reported complete resolution without intervention in a substantial proportion of cases at six-month follow-up [3]. As our study did not include systematic follow-up data, we cannot confirm this course in our own cohort; however, based on existing literature, routine clinical follow-up may be reasonable in asymptomatic or mildly symptomatic patients, with specific treatment reserved for associated conditions.

As a secondary, hypothesis-generating observation, *Giardia lamblia* stands out among associated conditions. Fecal calprotectin levels were significantly higher in *G. lamblia*–positive patients; however, the median value (36.5 µg/g) remained below the clinically accepted threshold (>50 µg/g; exceeded in 4 of the 10 *G. lamblia*–positive patients), suggesting that although the difference was statistically significant, its clinical relevance may be limited. This observation is based on only 10 patients, and in the absence of a control group, it cannot be determined whether the elevation reflects an NLH-specific process or simply the inflammatory effect of giardiasis itself. Given the small sample size and the number of comparisons performed without correction, a chance finding cannot be excluded. Moreover, multivariable adjustment was not feasible owing to the small number of patients; although age, sex, endoscopic ileitis, and nonspecific inflammation did not differ significantly between the groups, other factors that may influence fecal calprotectin, such as medication use, were not systematically recorded, and residual confounding cannot be ruled out. Nevertheless, *G. lamblia* infection has been reported in association with NLH in both immunodeficient and immunocompetent individuals [1,7,8,9,10,15,16], which may point to a broader role for chronic antigenic stimulation. Notably, three of the four patients with ileal ulcers were *G. lamblia*–positive, suggesting that mucosal injury in these patients may reflect accompanying infection-related ileitis rather than an NLH-specific process.

Beyond individual pathogens, alterations in gut microbiota have also been implicated in NLH; Jiang et al. reported dysbiosis characterized by decreased Bacteroidetes and increased Streptococcus, Fusobacterium nucleatum, and Actinomyces [17]. Although the gut microbiota was not assessed in our study, these findings are consistent with a potential role for chronic microbial stimulation in the pathogenesis.

A substantial proportion of patients in our series were active smokers (51.2%). Smoking has been shown to affect gut-associated lymphoid tissue in the terminal ileum, particularly Peyer’s patches, through increased epithelial apoptosis and immune cell accumulation [18]. For comparison, the prevalence of daily tobacco use among individuals aged ≥15 years in Turkey was 28.3% in 2022 (41.3% in men and 15.5% in women) [19]; the higher proportion in our series may partly reflect its male predominance and age structure, and no comparison with an age- and sex-matched local reference population was performed. Given the descriptive, cross-sectional design without a control group, no inference regarding a causal role of smoking in terminal ileal NLH can be drawn.

Celiac serology was negative in all patients, and no patient had serological evidence of celiac disease in this series. Although celiac disease has been reported in association with NLH in the literature, this association has been described predominantly in duodenal cases; however, in the absence of a control group and systematic duodenal biopsy, no conclusion can be drawn from our data regarding the relationship between celiac disease and terminal ileum–localized NLH.

NLH has frequently been reported in association with immunodeficiency states in the literature—particularly common variable immunodeficiency (CVID) and selective IgA deficiency [7,20,21,22]. In our series, however, no patient showed hypogammaglobulinemia or an immunoglobulin profile suggestive of immunodeficiency; IgA, IgG, and IgM levels were not below the lower reference limit in any patient. This finding is consistent with previous reports of NLH in patients with normal serum immunoglobulins, such as those with giardiasis [8], and indicates that terminal ileal NLH can also occur in adults without overt immunodeficiency. However, our assessment relied solely on baseline immunoglobulin levels, and mild or subclinical immunodeficiency cannot be excluded. Whether the low frequency of immunodeficiency in our series reflects the selection criteria of our cohort or a distinct etiopathogenesis of terminal ileum–localized NLH compared with duodenal/proximal forms remains unclear. Nonetheless, immunological evaluation may still be warranted in patients with recurrent infections, widespread gastrointestinal involvement, or unexplained laboratory abnormalities.

The principal strength of this study is the comprehensive evaluation of clinical, laboratory, endoscopic, and histopathological findings for this entity within the same series. The association between *Giardia* infection and elevated calprotectin should be regarded as an exploratory observation warranting further investigation.

Several limitations should be acknowledged. The retrospective, single-center design without a control group carries a risk of selection bias and limits our ability to determine whether the findings regarding associated conditions are specific to NLH. Moreover, because only patients who underwent colonoscopy with terminal ileal biopsy were included, the cohort may not be representative of all adults with terminal ileal NLH, particularly those with mild or asymptomatic disease in whom colonoscopy or biopsy is not performed. In addition, the total number of colonoscopies performed, the rate of terminal ileal intubation, and the number of patients with terminal ileal nodularity who did not undergo biopsy during the study period could not be retrieved from the records; therefore, the frequency of NLH among all colonoscopies could not be estimated. The sample size provides limited statistical power, particularly for subgroup comparisons; as no correction for multiple comparisons was applied and the *G. lamblia*–calprotectin association was not adjusted for potential confounders, the *p* = 0.016 value should be interpreted cautiously and confirmed in independent cohorts. Furthermore, seven patients with histologically confirmed NLH were excluded because of missing laboratory data, and a formal comparison between included and excluded patients was not performed; this may have introduced additional selection bias.

Histopathological re-evaluation was performed by a single pathologist, and interobserver agreement could not be assessed. Because the distinction between NLH, an isolated Peyer’s patch, and the lymphoid hyperplasia of early Crohn’s disease is partly subjective, single-observer assessment may have introduced systematic misclassification, which could have affected both patient inclusion and the frequency of histological findings; independent review by additional pathologists would be valuable in future studies. Data on antiparasitic treatment, pathogen clearance, and the subsequent clinical, endoscopic, or histological course were also not available; therefore, the findings regarding *G. lamblia* are limited to co-occurrence and an exploratory difference in fecal calprotectin, and neither the specificity of this association for NLH nor a causal relationship can be established. The number of biopsy specimens was not standardized, which may have affected both the rate of histopathological confirmation and the detection of less common findings. Gut microbiota and long-term clinical follow-up were beyond the scope of this study. Histopathological assessment relied solely on routine H&E morphology without immunohistochemistry or clonality testing; therefore, low-grade lymphoproliferative disorders cannot be definitively excluded. Accordingly, the absence of lymphoma in this small series should not be interpreted as evidence that lymphoma does not occur in association with NLH. Systematic follow-up data were also unavailable; although one patient was subsequently diagnosed with Crohn’s disease according to available records, later development of lymphoma or Crohn’s disease in other patients cannot be excluded. In addition, because histological confirmation was an inclusion criterion and no treatment decisions or clinical outcomes were analyzed, the diagnostic accuracy of endoscopy or histopathology and the effect of biopsy on management could not be evaluated. Future multicenter, prospective studies with control groups are needed to confirm these findings.

## 5. Conclusions

This study provides a comprehensive descriptive characterization of adult terminal ileal nodular lymphoid hyperplasia by evaluating clinical, laboratory, endoscopic, and histopathological findings together within the same series. Within the limits of routine histological assessment, our findings support the view that NLH is a predominantly benign and reactive lesion. In this series, histology confirmed NLH in 78.1% of patients biopsied on endoscopic suspicion, indicating that endoscopic appearance alone was insufficient for diagnosis in approximately one fifth of cases. Neither the histological demonstration of NLH nor isolated Crohn-suggestive features on biopsy can by themselves establish or exclude Crohn’s disease; diagnosis should therefore rely on clinicopathological correlation integrating clinical, endoscopic, laboratory, and histological findings, with follow-up when suggestive features are present. In addition, an exploratory association was observed between *G. lamblia* infection and elevated fecal calprotectin levels, which warrants confirmation in future studies. Larger, multicenter, prospective studies are needed to clarify these findings.

## Figures and Tables

**Figure 1:**
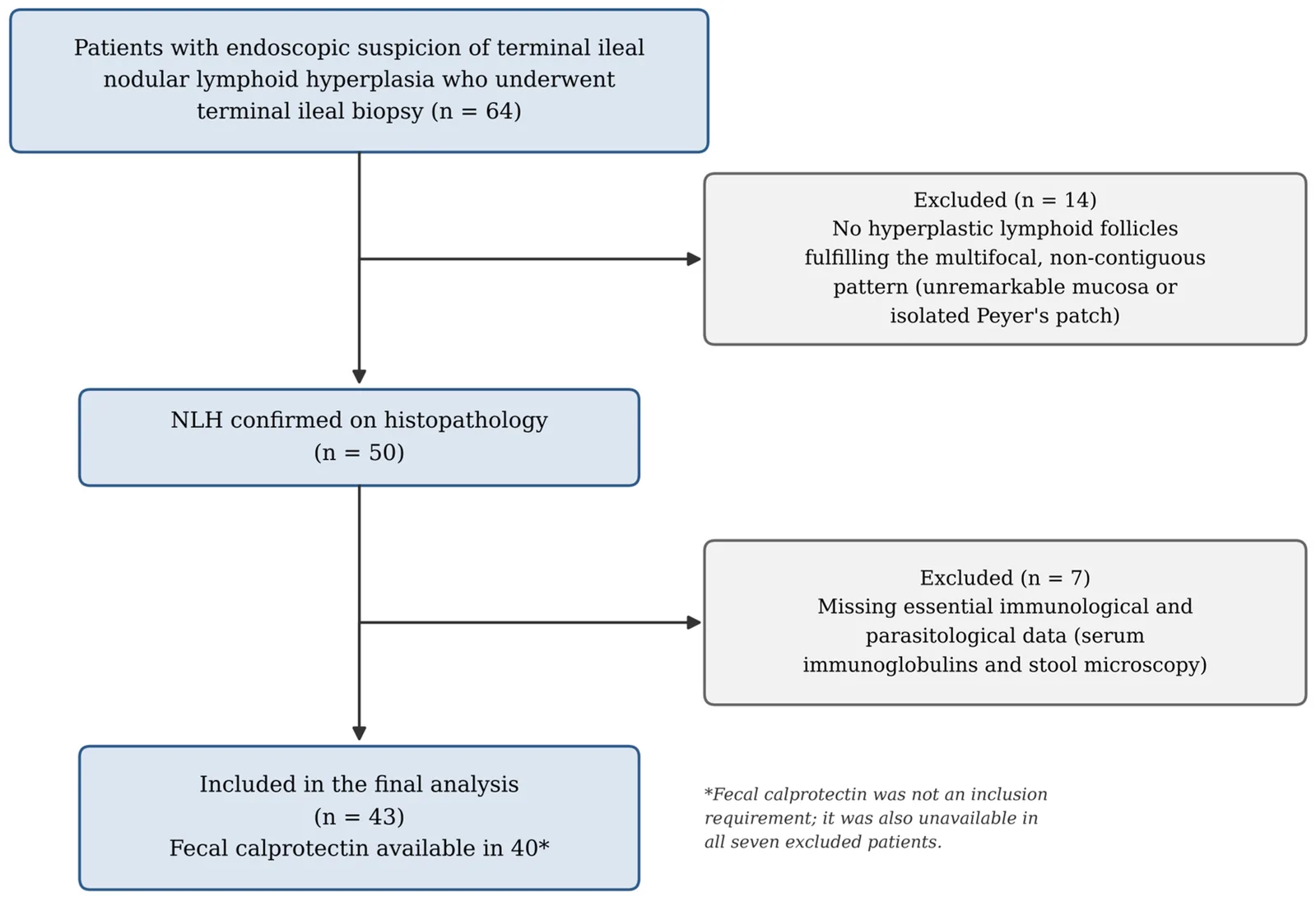
Flow diagram of patient selection.Blue boxes indicate patients retained in the analysis; gray boxes indicate excluded patients.

**Figure 2:**
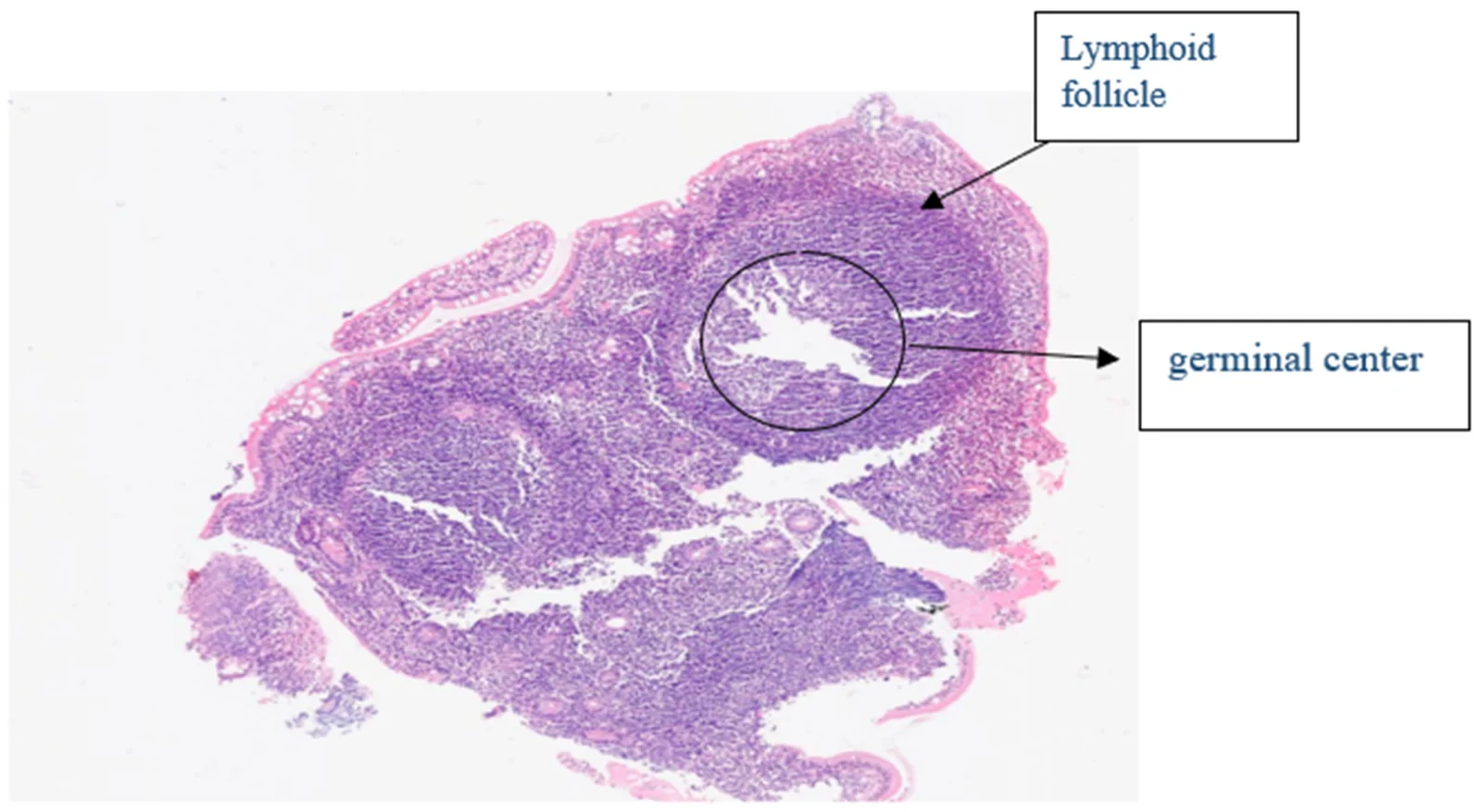
Histological section of the ileal mucosa showing nodular lymphoid hyperplasia, characterized by increased numbers of enlarged lymphoid follicles with prominent germinal centers within the lamina propria. Arrows indicate a representative lymphoid follicle and its germinal center (circled) (H&E, ×2).

**Figure 3:**
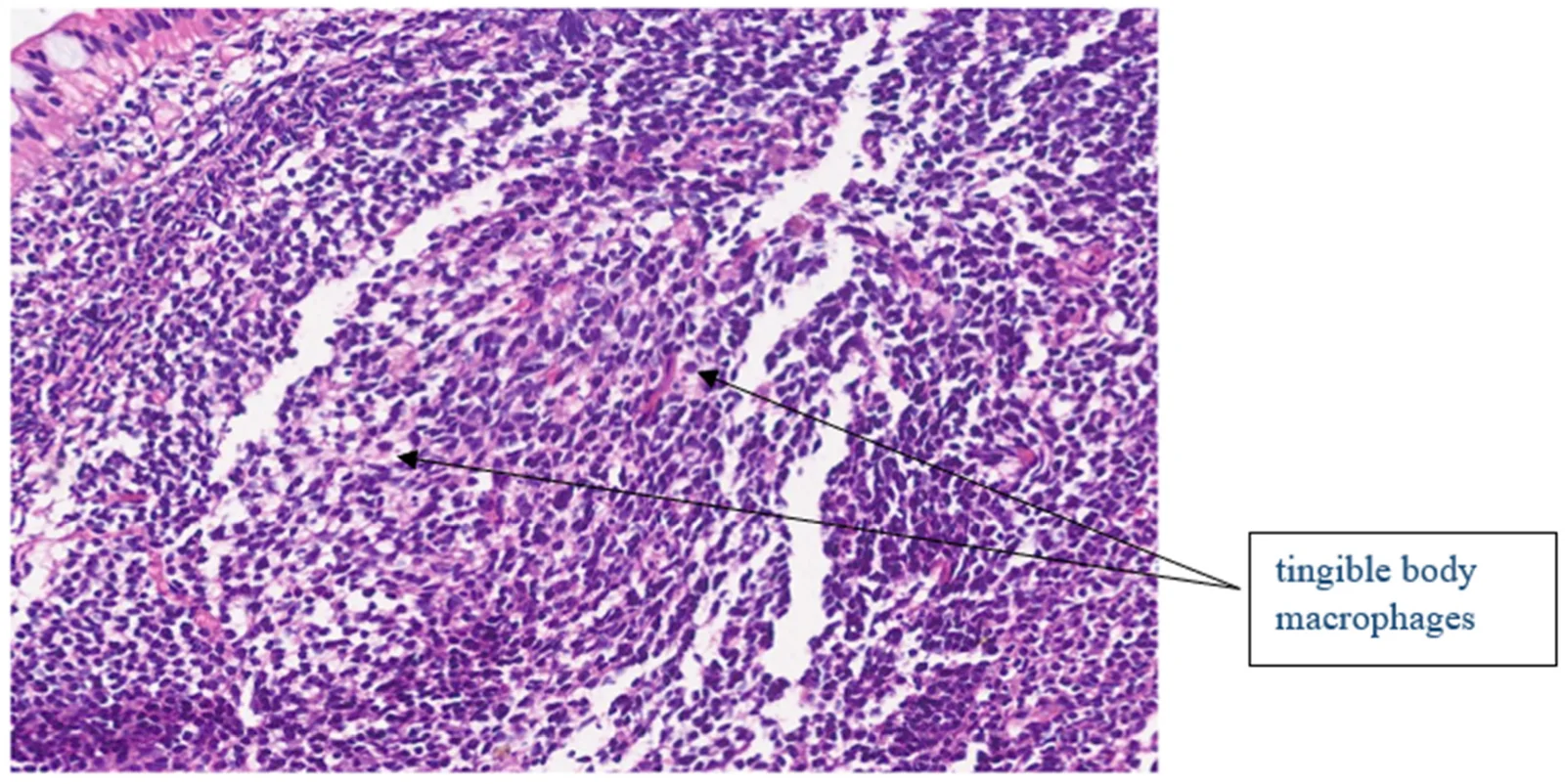
High-power view showing the morphologically heterogeneous lymphoid cell population composing the follicle, with tingible body macrophages within the germinal center (arrows) (H&E, ×10).

**Figure 4:**
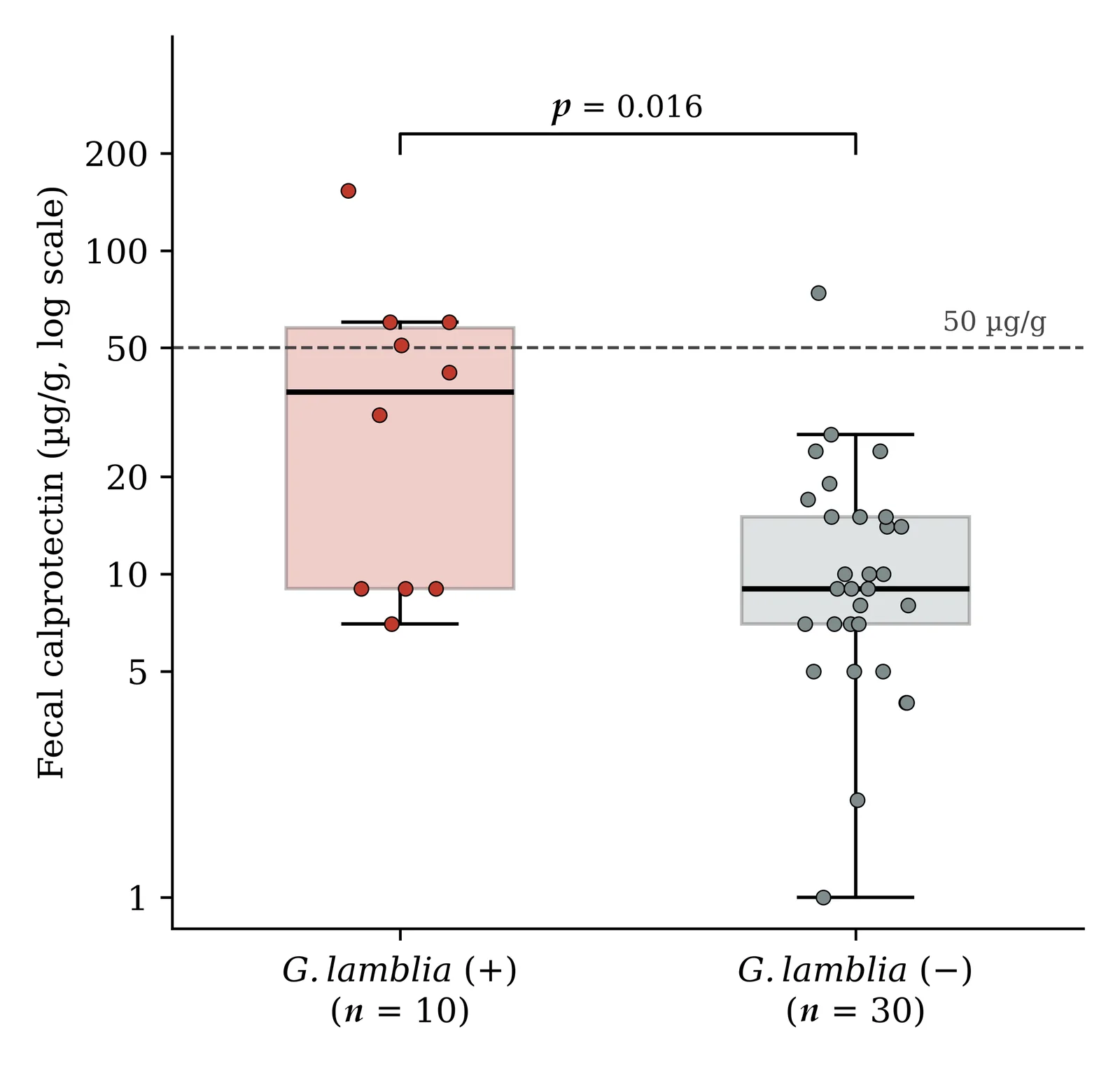
Fecal calprotectin level according to *G. lamblia* status. Boxes indicate medians and interquartile ranges, whiskers extend to 1.5× the interquartile range, and dots represent individual patients; the dashed line indicates the 50 µg/g threshold. The y-axis is on a logarithmic scale. Comparison by Mann–Whitney U test. Red indicates *G. lamblia*–positive and gray indicates *G. lamblia*–negative patients.

**Table 5:** Comparison of clinical, endoscopic, histopathological, and laboratory characteristics between *G. lamblia*–positive and *G. lamblia*–negative patients (secondary exploratory analysis).

Variable	*G. lamblia* (+) (*n* = 10)	*G. lamblia* (−) (*n* = 33)	*p*-Value
Age, years, median (IQR) ^a^	30.0 (23.3–40.0)	35.0 (24.0–43.0)	0.342 ^b^
Male sex, *n* (%)	6 (60.0)	23 (69.7)	0.704 ^c^
Endoscopic ileitis, *n* (%)	4 (40.0)	7 (21.2)	0.248 ^c^
Nonspecific histological inflammation, *n* (%)	10 (100.0)	29 (87.9)	0.558 ^c^
Fecal calprotectin, µg/g, median (IQR) ^d^	36.5 (9.0–57.8) (*n* = 10)	9.0 (7.0–15.0) (*n* = 30)	0.016 ^b^
Fecal calprotectin >50 µg/g, *n* (%)	4/10 (40.0)	1/30 (3.3)	0.010 ^c^

*G. lamblia* (+), *Giardia lamblia*–positive; *G. lamblia* (−), *Giardia lamblia*–negative. IQR, interquartile range. ^a^ Age was not normally distributed (Shapiro–Wilk test). ^b^ Mann–Whitney U test. ^c^ Fisher’s exact test. ^d^ Fecal calprotectin was available in 40 of 43 patients; the 3 patients without fecal calprotectin measurement were all *G. lamblia*–negative.

## Data Availability

The data presented in this study are not publicly available due to institutional confidentiality restrictions on patient records. Data may be made available by the corresponding author on reasonable request, subject to institutional approval.
